# Effect of 0.20% Beryllium (Be)-Added CuAl_10_Ni_5_Fe_4_ Alloy on Tribological Behavior and Microstructural Properties After Post-Casting Heat Treatment and Forging Process

**DOI:** 10.3390/ma17235757

**Published:** 2024-11-25

**Authors:** Khaled A. A. Babay, Ismail Esen, Selami Sagiroglu, Hayrettin Ahlatci, Esma Keskin

**Affiliations:** 1 Mechanical Engineering Department, Karabuk University, 78050 Karabuk, Turkey; 2138169055@ogrenci.karabuk.edu.tr (K.A.A.B.); ssagiroglu@karabuk.edu.tr (S.S.); 2 Metallurgical and Materials Engineering Department, Karabuk University, 78050 Karabuk, Turkey; hahlatci@karabuk.edu.tr; 3 BUMA Engineering and Consulting Inc., 06980 Ankara, Turkey; keskinesma5@gmail.com

**Keywords:** CuAl_10_Ni_5_Fe_4_, beryllium, heat treatment, forging process, tribological behavior, microstructure

## Abstract

This study explored how post-casting heat treatment and forging affected the tribological and microstructural characteristics of 0.20% beryllium (Be)-added CuAl_10_Ni_5_Fe_4_ alloys. The heat-treated CuAl_10_Ni_5_Fe_4_ microstructure exhibits a copper-rich α (alpha)-solid-solution phase, a martensitic β (beta)-phase, and diverse intermetallic κ (kappa)-phases, such as leaf-shaped κ_I_, thin κ_III_, and black globs. Adding 0.20% beryllium to CuAl_10_Ni_5_Fe_4_ alloys enhanced the dendritic arm thickness, needle-like shape, and κ-phase quantities. Significant κ_IV_- and κ_II_-phase precipitation was observed in the tempered β-phase. Beryllium improves the aluminum matrix’s microstructure. Forging greatly reduced the microstructural thickness of CuAl_10_Ni_5_Fe_4_ and CuAl_10_Ni_5_Fe_4_-0.20% Be alloys. The forging process also developed new κ_IV_-phases. Wear resistance and hardness improved with beryllium. The CuAl_10_Ni_5_Fe_4_-0.20% Be alloy had the highest hardness values (235.29 and 255.08 HB) after solution treatment (ST) and tempering (T) after casting and forging (F). The CuAl_10_Ni_5_Fe_4_-0.20% alloy with Be added had the best wear after solution treatment, tempering, and forging. The CuAl_10_Ni_5_Fe_4_-0.20% Be alloy demonstrated a 0.00272 g weight loss, a 1.36 × 10^−8^ g/N*m wear rate, and a 0.059 friction coefficient at 10,000 m after forging (F).

## 1. Introduction

The history of bronze alloys is a complex narrative that spans several millennia, reflecting advancements in metallurgy and the diverse applications of these materials across different cultures. Bronze, primarily an alloy of copper and tin, emerged during the Bronze Age, marking a significant technological leap from the use of pure copper. The earliest known bronzes were primarily tin bronzes, which were developed around 3500 BCE in regions such as the Near East and later in Europe and Asia. The introduction of tin not only improved the hardness and durability of copper but also expanded the range of applications for bronze, including tools, weapons, and decorative items [1,2,3]. Nickel–aluminum bronze (CuAl_10_Ni_5_Fe_4_) alloys are a significant class of copper-based alloys characterized by their unique compositions and properties, making them suitable for various applications, particularly in marine environments. The formation of CuAl_10_Ni_5_Fe_4_ alloys typically involves the addition of nickel, iron, and, sometimes, manganese to a copper–aluminum base. This combination results in a complex microstructure that includes various phases, such as α, β′, and κ, which contribute to the alloy’s mechanical strength and corrosion resistance [4,5,6]. The mechanical properties of CuAl_10_Ni_5_Fe alloys are enhanced through specific manufacturing processes, including casting, heat treatment, and additive manufacturing techniques. For instance, studies have shown that heat treatment can significantly improve the microstructure and mechanical properties of CuAl_10_Ni_5_Fe alloys by refining the grain size and promoting phase transformations [5,7] to produce NAB components with superior mechanical properties, demonstrating the versatility of these alloys in modern manufacturing [8,9]. The ability to manipulate the microstructure through these processes is crucial, as it directly influences the performance of CuAl_10_Ni_5_Fe_4_ in demanding applications, such as ship propellers and underwater fasteners [8]. The microstructural evolution of the CuAl_10_Ni_5_Fe alloy is a complex process influenced by various factors, including the cooling rate, phase transformations, and the presence of alloying elements. This alloy, primarily composed of copper, aluminum, nickel, and iron, exhibits a unique microstructure characterized by a combination of different phases, which significantly affect its mechanical properties and wear and corrosion resistances. Initially, during the solidification process, the CuAl_10_Ni_5_Fe alloy transitions from a liquid to a solid state, where the β-phase, a body-centered-cubic (BCC) structure, forms at high temperatures. This phase is crucial, as it serves as the primary solid phase before the alloy cools down to lower temperatures, specifically around 1030 °C, where the α-phase, a face-centered-cubic (FCC) structure, begins to develop from the β-phase [10]. The presence of intermetallic κ-phases also contributes to the overall microstructure, which can influence this alloy’s mechanical properties and its resistance to wear and corrosion.

The tribological properties of the nickel–aluminum bronze alloy CuAl_10_Ni_5_Fe have garnered significant attention because of its applications in marine environments, particularly in ship propellers, pumps, and valves. This alloy is recognized for its excellent corrosion resistance and mechanical properties, making it a preferred choice in naval applications. The study of its tribological characteristics is crucial, as it can provide insights into its performance under various operational conditions. CuAl_10_Ni_5_Fe_4_ exhibits superior wear resistance and anti-friction properties compared to those of other alloys. Tan et al. highlight that this alloy has been developed to enhance the strength and wear resistance of traditional aluminum bronzes, which are already well regarded for their mechanical properties and resistance to marine bio-fouling [11]. This alloy’s performance is particularly notable when subjected to corrosion treatments, which can significantly influence its tribological behavior. Research indicates that understanding the wear mechanisms and the factors affecting these properties is essential for optimizing this alloy’s use in demanding environments [11]. Heat treatment processes also play a pivotal role in enhancing the tribological characteristics of CuAl_10_Ni_5_Fe_4_. Yaseen et al. investigated the effects of various heat treatments, including annealing, quenching, and aging, on this alloy’s microstructure and tribological performance. Their findings suggest that these treatments can significantly alter the microstructural features of the alloy, which, in turn, affect its wear resistance and frictional properties [12]. The correlation between the microstructure and tribological performance emphasizes the importance for processing techniques in maximizing the utility of CuAl_10_Ni_5_Fe_4_ in high-wear applications. Research indicates that CuAl_10_Ni_5_Fe_4_ exhibits a lower coefficient of friction compared to those of other aluminum bronzes, which enhances its wear resistance under certain conditions [13]. This alloy’s microstructural characteristics, including the presence of solid intermetallic inclusions, play a significant role in its tribological behavior. These inclusions can create a beneficial microrelief on the friction surface, reducing wear [14]. Additionally, the incorporation of alloy elements has been shown to further enhance the corrosion and wear resistances of CuAl_10_Ni_5_Fe_4_, particularly at elevated temperatures [15]. This modification can lead to improved mechanical properties, making this alloy even more suitable for demanding applications.

The incorporation of beryllium into copper-based alloys, such as CuAl_10_Ni_5_Fe_4_, enhances mechanical properties, including hardness and wear resistance, which are essential for components subjected to high-stress conditions. For instance, the presence of beryllium in copper enhances this alloy’s strength and wear resistance through the formation of stable γ-precipitates, which contribute to the overall hardness of the material [16,17]. These alloys further optimize their wear performance [17,18]. This is particularly relevant for applications in aerospace and automotive industries, where components must endure extreme conditions without significant degradation [19]. Moreover, the addition of beryllium (Be) to the CuAl_10_Ni_5_Fe_4_ alloy significantly influences its microstructural and mechanical properties. Beryllium is known for its ability to enhance the strength and hardness of copper-based alloys, including those with aluminum, nickel, and iron. The microstructural evolution in these alloys typically involves the formation of specific phases that contribute to improved performance characteristics. In the context of beryllium-containing alloys, the precipitation of the NiBe phase has been documented, particularly in nickel–chromium alloys, where the presence of beryllium leads to the formation of distinct microstructural features [20]. This phenomenon is also relevant in copper-based alloys, where beryllium can precipitate as a phase that enhances the mechanical properties by refining the microstructure [21]. The equilibrium phase (γ), precipitated by beryllium and nickel, has been shown to grow predominantly at grain boundaries during aging, which can lead to inhomogeneous properties across the alloy [21]. Heat treatment cycles are critical for achieving peak hardness and strength in copper–beryllium alloys, as they allow for the optimal precipitation of strengthening phases [16,22,23,24].

The microstructural refinement associated with beryllium addition contributes to a more uniform distribution of phases, which can mitigate wear mechanisms, such as adhesive and abrasive wear [25]. Moreover, the tribological characteristics of beryllium-containing alloys have been extensively studied, revealing that they exhibit favorable wear resistance under various loading conditions. The wear performance of CuAl_10_Ni_5_Fe_4_, particularly when subjected to sliding conditions, can be optimized by adjusting the beryllium content. Research has shown that beryllium-containing copper alloys demonstrate lower coefficients of friction and reduced wear volumes compared to their non-beryllium counterparts, which is essential for prolonging the service life of components [26,27]. Furthermore, the interaction between an alloy’s microstructure and its wear performance is critical. The addition of beryllium not only enhances mechanical properties but also influences the alloy’s response to heat treatment, which can further improve wear resistance. Heat treatment processes can lead to precipitation hardening, which is beneficial for enhancing the hardness and wear resistance of the alloy [16,28].

In conclusion, the addition of beryllium to CuAl_10_Ni_5_Fe_4_ alloys significantly influences their microstructural evolutions, leading to improvements in mechanical properties, tribological characteristics, and corrosion resistances. The formation of intermetallic phases, the refinement of the precipitate’s morphology, and the enhancement of passive layers are key factors that contribute to the overall performance of these alloys in various applications. This makes beryllium-modified CuAl_10_Ni_5_Fe_4_ alloys significantly enhanced through appropriate alloying and treatment processes and highly suitable for applications requiring high wear resistance and durability. In this study, it was investigated how the tribological and microstructural properties of 0.20% beryllium (Be)-added CuAl_10_Ni_5_Fe_4_ alloys were affected by post-casting heat treatment (solution treatment (ST) and tempering (T)) and forging (F) methods.

### The Novelty of This Study

Although the addition of beryllium to CuAl_10_Ni_5_Fe_4_ has a significant advantage in improving wear properties, the effects of minor amounts of beryllium added to CuAl_10_Ni_5_Fe_4_ on the tribological performance have not been investigated. In addition, the lack of research on fundamental studies, such as microstructures and alloy phases, has created a gap in the literature. In this study, how minor amounts of beryllium added to CuAl_10_Ni_5_Fe_4_ change the microstructural and tribological properties of the alloy has been investigated in detail, and the results have been reported. Therefore, this study aimed to provide high performance for harsh environments, such as marine and aerospace applications, where the tribological resistance of CuAl_10_Ni_5_Fe_4_ alloys is desired.

## 2. Materials and Methods

The casting experiments for the production of samples were carried out at the R&D Center of Saglam Metal Industry and Trade (Kocaeli, Turkey). An already produced ingot made of nickel–aluminum bronze was obtained for the purpose of using it as a raw material in casting trials, in accordance with manufacturing criteria. Ingots produced in industrial settings were used as the main material, while materials specifically designed for laboratory study were created. The ingot, utilized as the primary material for conducting casting tests, was produced using the permanent mold-casting technique and comprised pure copper scrap, pure aluminum, iron, and nickel. The melting procedure was carried out utilizing an induction furnace with a 300 kg capacity. The fabricated nickel–aluminum bronze ingot was cut and used for the process of melting. The CuLi pre-alloy was used to remove oxygen from the liquid metal, while a 30% copper–beryllium pre-alloy was used to add beryllium. The process for removing gases after melting nickel–aluminum bronze involves the utilization of a copper–lithium pre-alloy. Two separate charges were created: one without beryllium and the other having 0.20% beryllium. Furthermore, a 30% copper–beryllium pre-alloy was added to the molten metal in the final stage of melting before the casting process began. The castings were performed at a temperature of 1230 °C. Table 1 displays the chemical compositions of the CuAl_10_Ni_5_Fe_4_ and CuAl_10_Ni_5_Fe_4_-0.20% Be alloys. The chemical compositions of the ingots, which were manufactured in an industrial setting, were analyzed using an Oxford brand optical emission spectrometer at the same facilities where the casting process took place. The alloys’ phases were identified using X-ray diffraction analysis using a Rigaku Ultima IV instrument from Japan. The scanning range was set from 10 to 90 degrees, and the scanning speed was 3 degrees per minute. The samples produced using the casting process underwent heat treatments after solution treatment (ST) at a temperature of 875 °C for a duration of 90 min and then tempering (T) at a temperature of 650 °C for a duration of 2 h.

Industrial conditions were utilized to perform experiments on forging using a ram. The forging conditions were set at a temperature of 870 °C [29], which is known to provide the highest strength values according to the literature at a deformation rate of 80% [30]. To accomplish this goal, cylindrical metal bars with a diameter of 68 mm underwent a forging process. This procedure involved employing a ram as a tool to transform the bars to a final section size of 27 × 27 mm.

CuAl_10_Ni_5_Fe_4_ and CuAl_10_Ni_5_Fe_4_-0.20% Be samples, which had been subjected to heat treatment and forging, were each divided into pieces of dimensions 10 × 20 × 10 mm. The cutting procedure was executed utilizing a band saw that was cooled using water. After completing the cutting operation, the samples were subjected to sanding and polishing using a Mikrotest brand automated apparatus (Mikrotest Co, Istanbul, Turkey). A range of grit sizes, such as 320, 400, 600, 800, 1000, and 2500, were utilized throughout the sanding process. Following the sanding process, the polishing was conducted using a liquid solution of Al_2_0_3_ with a particle size of 3 μm. The etching process included 5 g of FeCl_3_, 50 mL of HCl, and 100 mL of distilled water. An LOM–Carl Zeiss light optical microscope (ZEISS Group, Darmstadt, Germany) was used to examine the changes in the grains within the phase structure. In addition, an SEM–Carl Zeiss Ultra Plus scanning electron microscope (ZEISS Group, Darmstadt, Germany) and EDX (energy-dispersive X-ray spectroscopy) were used to investigate and identify any secondary phases. Steel balls of 2.5 mm in diameter were exposed to a force of 187.5 N to determine their Brinell hardness.

Tribological testing was conducted at ambient temperature, with the loading axis aligned parallel to the rolling direction (L). Rectangular-prism-shaped samples measuring 18 mm by 14 mm by 10 mm were manufactured for wear testing. Before the abrasion test, the determined surfaces of the sample were smoothed using 1200 μm sandpaper. Then, the samples cleaned with ethanol were weighed on a Precisa brand balance (Precisa Gravimetrics AG, CH-8953 Dietikon, Switzerland) with a precision of 0.1 mg. The abrasion tests were carried out under dry conditions by applying a 20 N load at a sliding speed of 0.1 m/s and by covering a total distance of 10,000 m using a back-and-forth abrasion tester. A high-hardness ball made of AISI-52100-quality steel was used as an abrasive tip. Ethanol was employed once more to clean the residual wear debris off the surfaces of the samples taken from the abrasion tester at intervals of every 200 m. The samples, cleaned with ethanol, were weighed on the precision scale before the back-and-forth abrasion test was resumed. Weight reductions, depending on the distance, were calculated cumulatively by subtracting the initial weight from the final weight using these data. The mass loss data were converted to a specific wear rate using Equation (1) as follows:

(1) $$Wear\;rate\;per\;unite\;force\left(\frac{g}{Nm}\right)=\frac{Mass\;loss\;due\;to\;wearing\;(g)}{Force\;Applied\;\left(N\right)\times Displacement(m)}$$

The friction force during wear was quantified utilizing a load cell connected to the tribometer arm and instantly documented on the computer. The wear mechanism was investigated by employing scanning electron microscopy (SEM) and energy-dispersive X-ray spectroscopy (EDX) tools to assess alterations in the concentrations of the alloy elements and the applied stress during the wear test.

## 3. Results and Discussion

### 3.1. XRD Patterns

**Figure 1:**
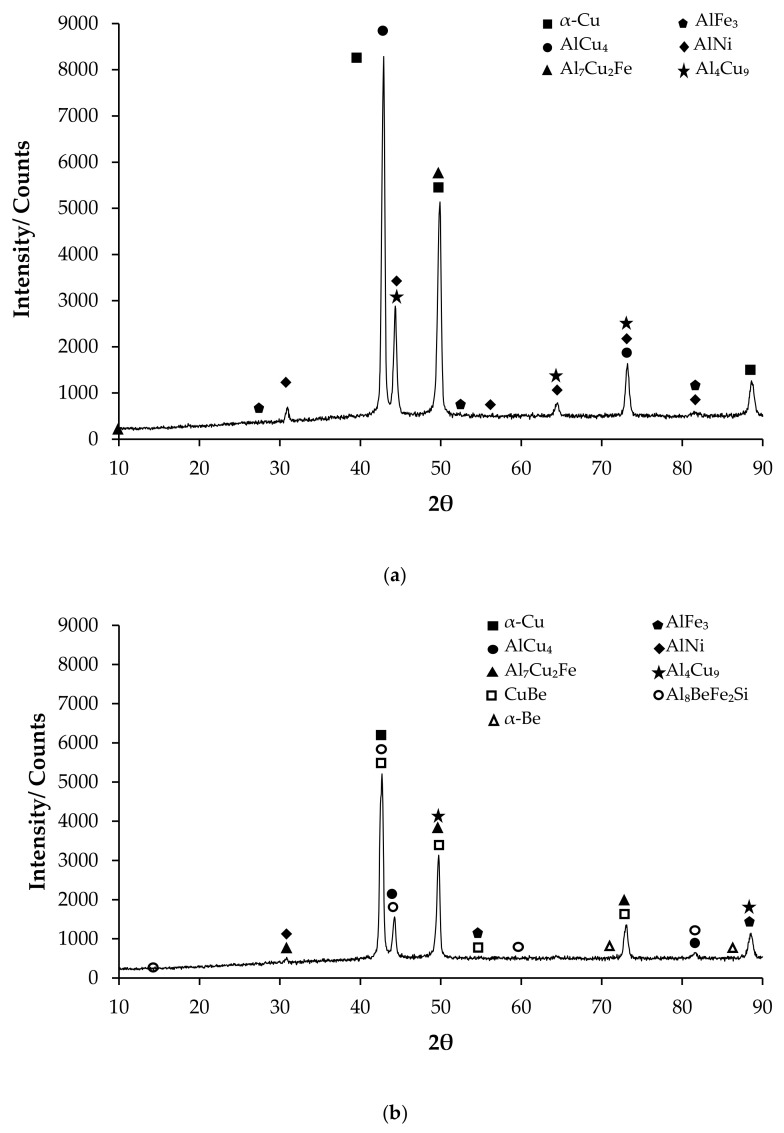
Patterns of X-ray diffraction (XRD): (**a**) CuAl_10_Ni_5_Fe_4_ and (**b**) CuAl_10_Ni_5_Fe_4_-0.20%Be.

X-ray diffraction (XRD) analysis is an essential technique in materials research, offering vital information on the crystallographic structure, phase composition, and microstructural properties of diverse materials. The importance of XRD is evident in several fields, including materials characterization, industrial quality control, and the development of novel materials [31]. XRD patterns enable the identification of materials with intricate compositions [32]. The CuAl_10_Ni_5_Fe_4_ alloy has enhanced wear resistance while maintaining ductility, which is attributed to the presence of κ-phases [10]. XRD studies presented in Figure 1 show that α-Cu phases are observed at the highest peak in both alloys, while CuBe and Al_8_BeFe_2_Si phases are also observed with the addition of beryllium. Figure 1a shows the first-phase peak of the CuAl_10_Ni_5_Fe_4_ alloy at a 13.50° angle as Al_7_Cu_2_Fe, and the last peak is α-Cu at 88°. Figure 1b shows that the CuAl_10_Ni_5_Fe_4_-0.20% Be alloy starts with the Al_8_BeFe_2_Si phase at a 22° angle, indicating the presence of beryllium. The last peak is AlFe_3_ and Al_4_Cu_9_ at 88.50°.

### 3.2. Microstructure

**Figure 2:**
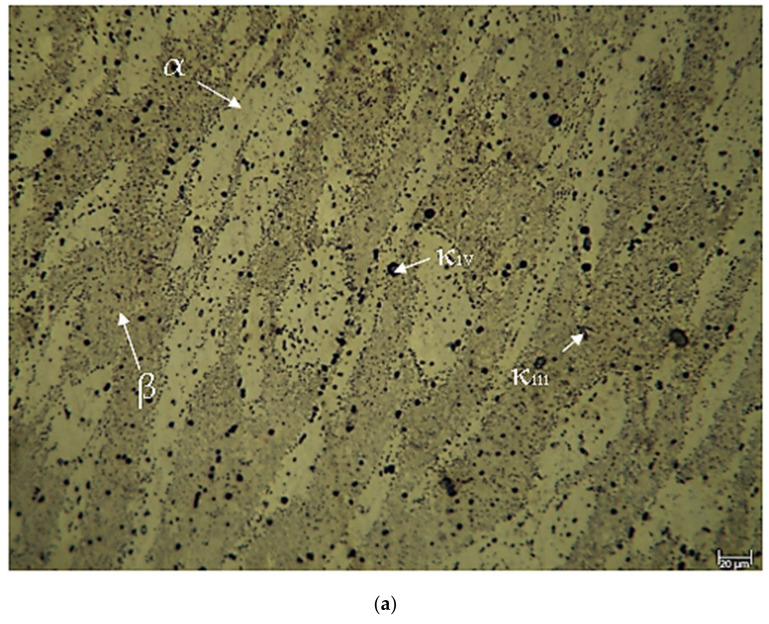

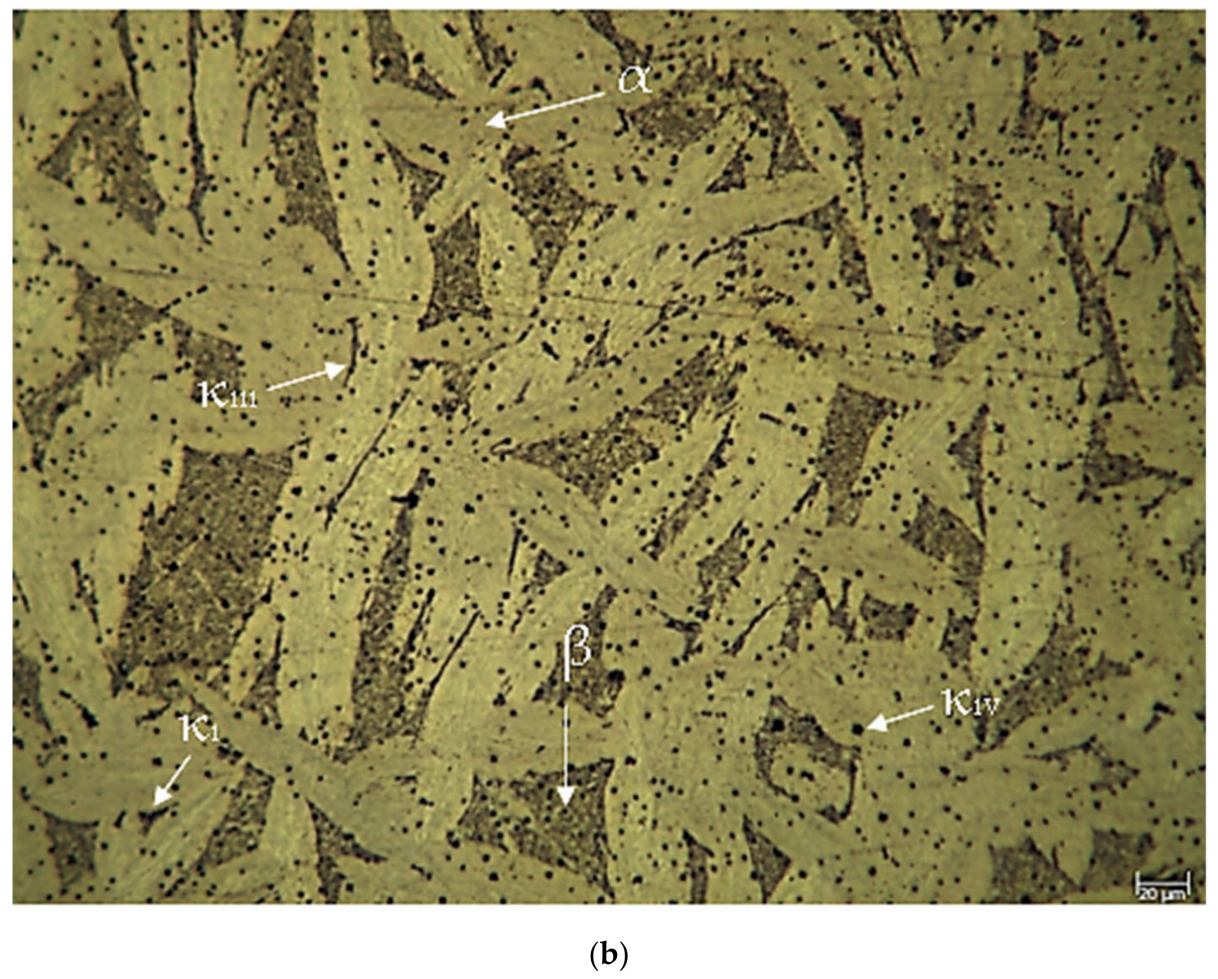
LOM images after ST and T after casting: (**a**) CuAl_10_Ni_5_Fe_4_ and (**b**) CuAl_10_Ni_5_Fe_4_-0.20% Be.

**Figure 3:**
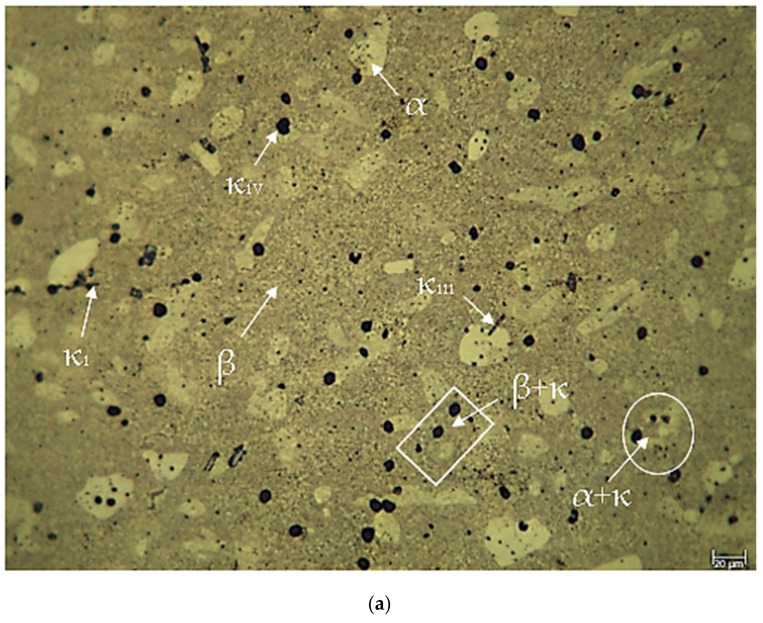

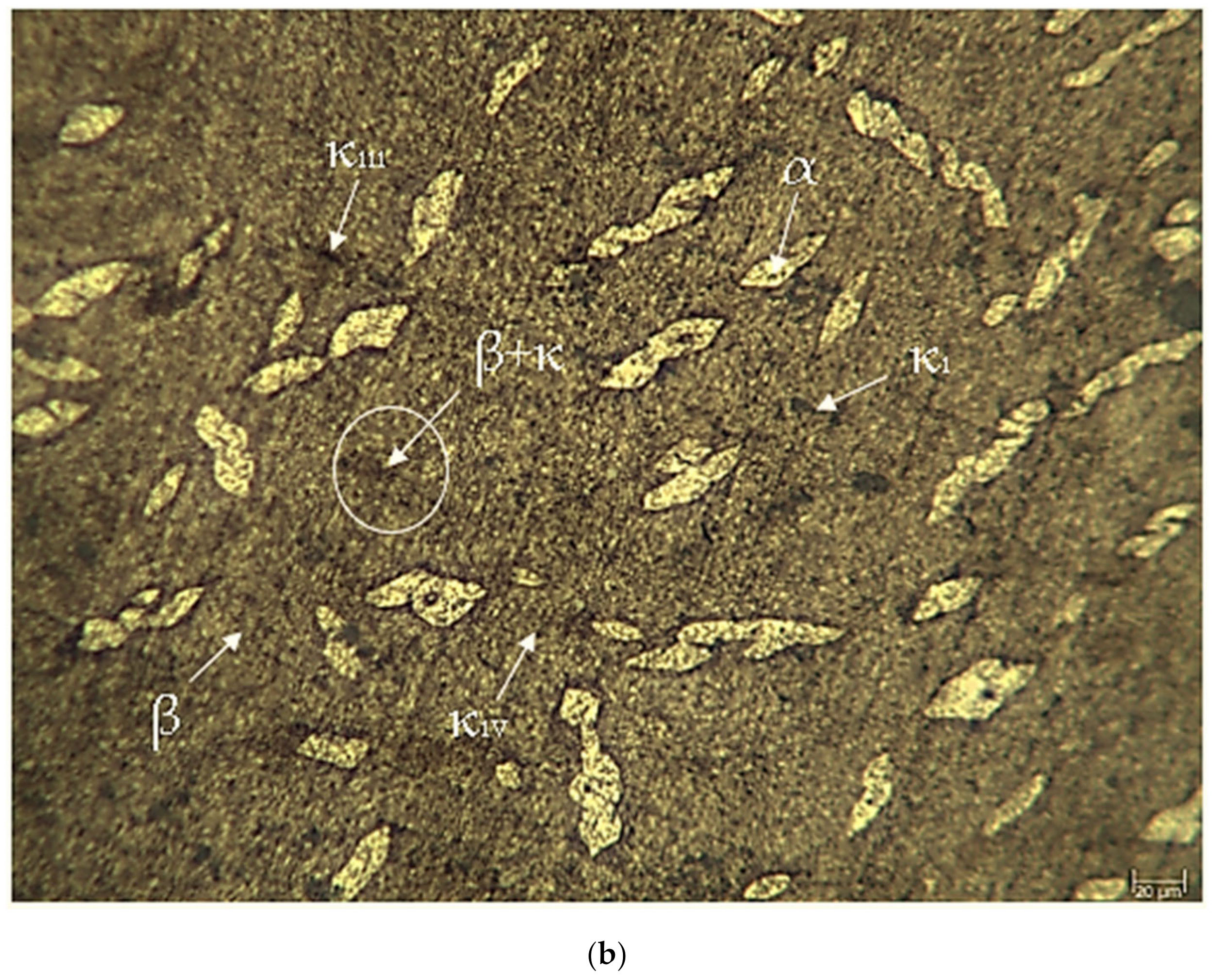
LOM images after F: (**a**) CuAl_10_Ni_5_Fe_4_ and (**b**) CuAl_10_Ni_5_Fe_4_-0.20% Be.

Figure 2 illustrates the LOM images of the CuAl_10_Ni_5_Fe_4_ and CuAl_10_Ni_5_Fe_4_-0.20% Be alloys, which should undergo solution treatment and tempering subsequent to casting. Figure 3 displays the LOM pictures of the identical alloys post forging. A factor that aids in grain refining upon the addition of elements is the enhancement of the grain formation rate before the proliferation of grain nuclei occurs. An augmentation in the quantity of nucleation sites per unit of volume results in the formation of smaller grains [33]. Beryllium is known to influence the microstructures of various alloys, including copper-based systems, by altering phase distributions and precipitate formations. Beryllium’s role in modifying microstructures can be observed in its interaction with the copper matrix. For instance, the addition of beryllium can lead to the formation of intermetallic phases that enhance mechanical properties. Specifically, in the context of copper alloys, the presence of beryllium has been shown to promote the formation of fine precipitates [21,28]. The microstructural characteristics, such as the size and distribution of these precipitates, are critical for the alloy. Studies indicate that the addition of beryllium can lead to a refined microstructure [25]. CuAl_10_Ni_5_Fe_4_ clearly demonstrates the presence of a copper-rich α (alpha)-solid-solution phase, a martensitic β (beta)-phase, and several intermetallic κ (kappa)-phases, including leaf-shaped κ_I_, thin κ_III_, and black globular κ_IV_ [12,34,35,36]. Figure 2b shows that the addition of 0.20% beryllium to CuAl_10_Ni_5_Fe_4_ alloys resulted in an increase in the thickness and the needle-like shape of the dendritic arms formed by α-phases, as well as increases in the amounts of certain κ-phases. Furthermore, significant κ_IV_- and κ_II_-phase precipitation was detected in the tempered β-phase [12]. Beryllium promotes a refined microstructure within the aluminum matrix [37,38]. Research indicates that the incorporation of beryllium leads to the formation of a more uniform distribution of phases within the aluminum bronze matrix. This is attributed to the ability of beryllium to modify the solidification behavior of the alloy, resulting in finer dendritic structures and reduced porosity [24,39]. The microstructural evolution is also influenced by the thermal treatment processes, where beryllium can affect phase transformations and the stability of the microstructure [38]. The transformation of the precipitate morphology from irregular shapes to more uniform rod-like structures has been documented [28]. The thickness of the microstructures in the CuAl_10_Ni_5_Fe_4_ and CuAl_10_Ni_5_Fe_4_-0.20% Be alloys significantly decreased during the forging process, as shown in Figure 3. The alloys maintained the same α-, β-, and κ-phases as those shown in Figure 2 following the forging procedure. Because of the forging process, the dendritic arms underwent fracturing and were subsequently converted to almost spherical forms. In addition, the forging process resulted in the creation of additional κ_IV_-phases. The addition of 0.20% beryllium to the CuAl_10_Ni_5_Fe_4_ alloy resulted in the formation of distinct κ_I_- and κ_III_-phases in the alloys.

**Figure 4:**
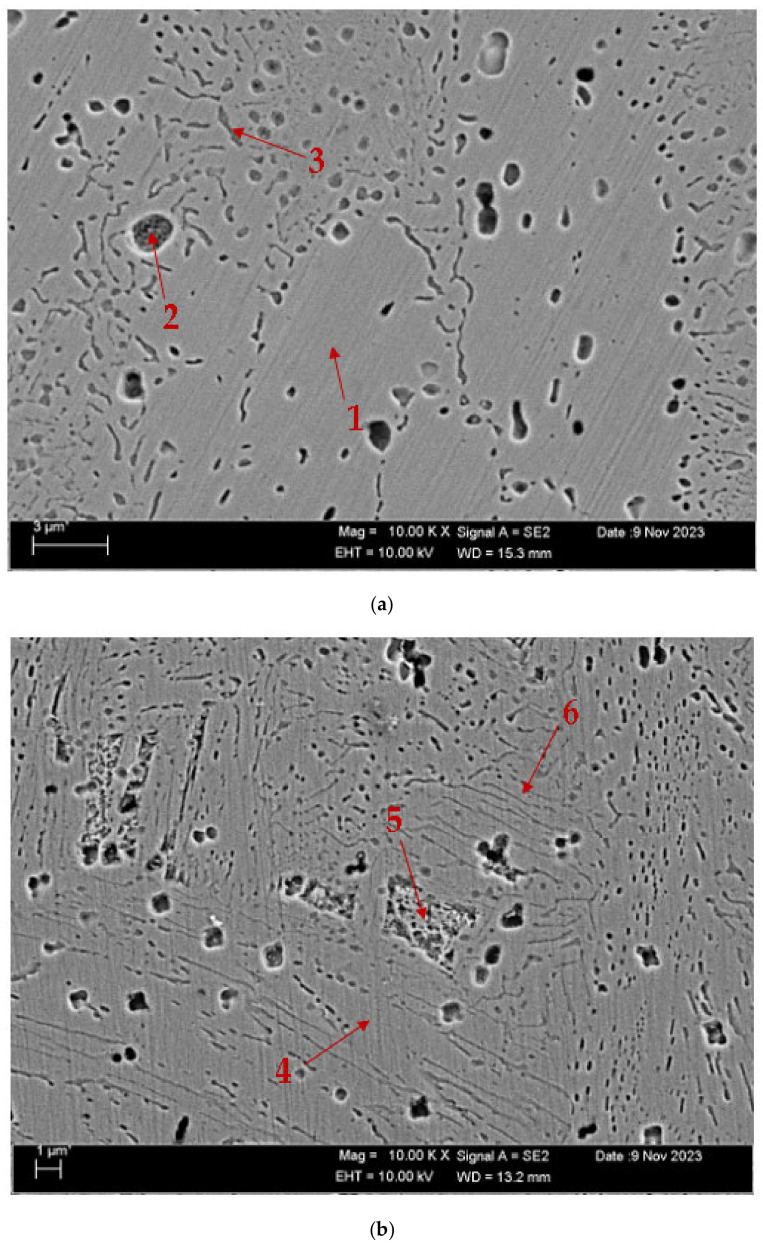
SEM images after ST and T after casting: (**a**) CuAl_10_Ni_5_Fe_4_ and (**b**) CuAl_10_Ni_5_Fe_4_-0.20% Be.

**Figure 5:**
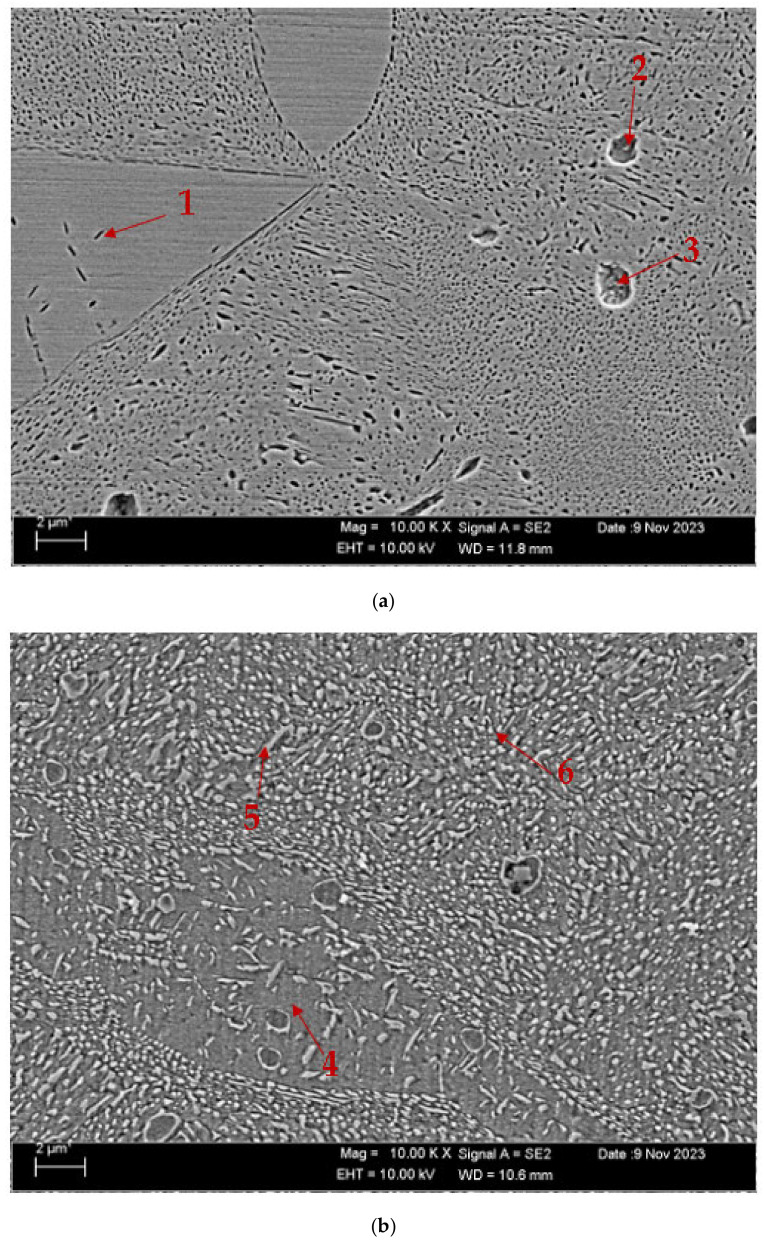
SEM images after F: (**a**) CuAl_10_Ni_5_Fe_4_ and (**b**) CuAl_10_Ni_5_Fe_4_-0.20% Be.

Figure 4 displays the scanning electron microscope (SEM) pictures of the CuAl_10_Ni_5_Fe_4_ and CuAl_10_Ni_5_Fe_4_-0.20% Be alloys following the solution treatment (ST) and tempering (T) processes after casting. On the other hand, Figure 5 exhibits the SEM images of the same alloys after the forging process (F). Table 2 and Table 3 show the EDX analysis results of the second phases, which exhibit different morphologies at the arrowhead points labeled as (1–6 in Figure 4a,b and Figure 5a,b, respectively. The SEM pictures provide a comprehensive view of the microstructure of the alloys, which comprise α-, β-, and a range of intermetallic κ-phases. The α-phase is a solid solution with a high concentration of copper, whereas the β-phase, also known as the retained β-phase, is a solid solution with a tempered structure and a dense region of AlNi precipitates. There exist three distinct κ-phases, characterized by their specific morphologies and distributions. The κ_I_-phase is characterized by a high concentration of iron and is located within the α-phase matrix, which usually exhibits a rosette-shaped morphology. The κ_IV_-phase is predominantly located near the boundary between the α- and β-phases, forming a shape resembling a sphere. The κ_IV_-phase is far more refined than the κ_I_-phase. The lamellar κ_III_-phase, characterized by a high nickel content, is formed at α- and β-phase borders by the eutectoid transition of the β-phase under low-temperature conditions. The κ_IV_-phase, characterized by a high iron content, is a more refined precipitate inside the α-phase [40,41,42].

### 3.3. Hardness Test Results

**Figure 6:**
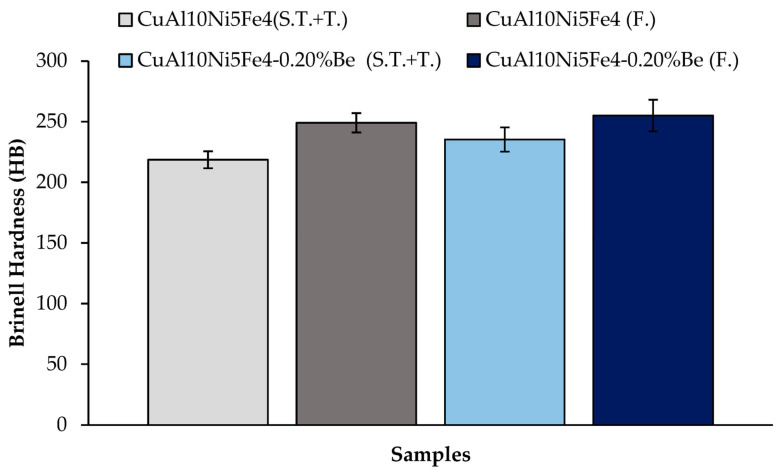
Hardness comparison of CuAl_10_Ni_5_Fe_4_ and CuAl_10_Ni_5_Fe_4_-0.20% Be alloys.

Figure 6 presents a comparative analysis of the hardness values of the CuAl_10_Ni_5_Fe_4_ and CuAl_10_Ni_5_Fe_4_-0.20% Be alloys. The analysis includes measurements taken after solution treatment (ST) and tempering (T) for both casting and forging processes. Although forging greatly increased the hardness of both alloys, the hardness of the CuAl_10_Ni_5_Fe_4_ alloy without the beryllium addition was observed to be lower. Considering the difference in the hardness values after the solution treatment (ST) and tempering (T) after casting and after forging (F), it was observed that the CuAl_10_Ni_5_Fe_4_-0.20% Be alloy showed the highest values of 235.29 HB and 255.08 HB, respectively.

The hardness of the nickel–aluminum bronze results from the intricate interaction of its microstructural phases, α, β, and κ. The β-phase substantially enhances hardness, owing to its martensitic properties, while the κ-phases augment strength through their intermetallic features. The κ-phase, including various intermetallic compounds generated after solidification, is crucial for augmenting the hardness of CuAl_10_Ni_5_Fe_4_. The inclusion of κ-phases can result in a more intricate microstructure that enhances wear resistance and strength [15,43]. The intermetallic characteristics of the κ-phases enhance the alloy’s total hardness, as these phases are generally harder than both the α- and β-phases. The distribution and form of these κ-phases may be affected by the alloying elements [44,45]. The forging process substantially affects material hardness, primarily through processes including grain refinement, phase transitions, and temperature impacts during deformation. Forging techniques, particularly when coupled with heat treatment, have demonstrated improvements in the mechanical and tribological characteristics of CuAl_10_Ni_5_Fe_4_, including the hardness, yield strength, and tensile strength. Hot forging at extreme temperatures, around 850 °C, has been shown to markedly enhance the yield strength and tensile strength of nickel–aluminum bronze while simultaneously diminishing elongation. This effect results from microstructural refinement during the forging process, which increases the dislocation density and alters the grain structure, hence enhancing hardness [46]. Furthermore, the hardness of CuAl_10_Ni_5_Fe_4_ can be also affected by the use of alloying materials [47]. Research indicates that altering alloy compositions might result in varied mechanical qualities, such as hardness [48]. Moreover, the incorporation of beryllium into copper-based alloys can lead to improvements in microstructural characteristics, such as grain refinement and the modification of phase distributions. This is particularly relevant in the context of CuAl_10_Ni_5_Fe_4_, where the presence of beryllium may facilitate the formation of a more uniform microstructure, thereby enhancing the overall hardness of the alloy. Studies have indicated that the microstructural evolution in beryllium–copper alloys can significantly impact their mechanical properties, including hardness [16,21,49]. This is primarily because of the formation of fine, hard precipitates that impede dislocation movement within the metal matrix, thereby increasing hardness and strength [50]. Nickel–aluminum bronzes are characterized by their complex microstructures, which include various phases, such as α and β’, and intermetallic compounds. The introduction of beryllium can modify these microstructures, potentially leading to improved mechanical properties. Specifically, the presence of beryllium can refine grain structures and enhance the distribution of strengthening phases, which contributes to increased hardness [50]. Research indicates that the hardness of CuAl_10_Ni_5_Fe_4_ alloys can be significantly influenced by the thermal history and processing conditions, including solidification rates and heat treatments. Beryllium’s role in these processes can further enhance the mechanical properties of the alloy. For instance, studies have demonstrated that the addition of beryllium can lead to a more uniform distribution of phases, which is beneficial for achieving optimal hardness levels [51]. Furthermore, the interplay between beryllium and other alloying elements, such as nickel and aluminum, is critical, as these interactions can lead to synergistic effects that enhance the overall performance of the alloy [4].

### 3.4. Tribological (Wear) Test Results

**Figure 7:**
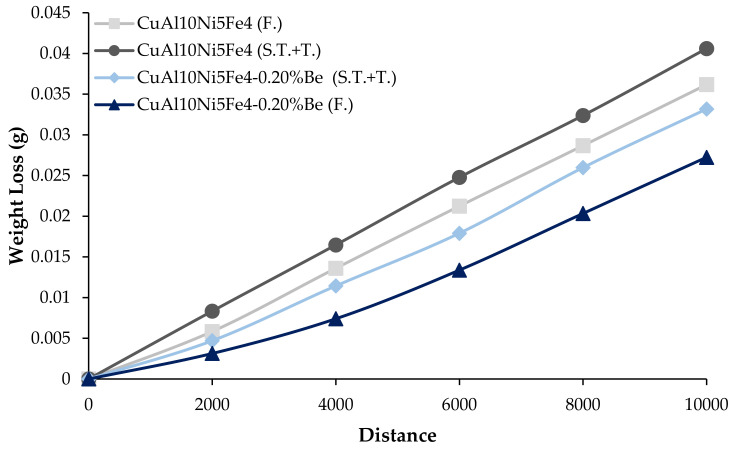
Weight loss with wear of CuAl_10_Ni_5_Fe_4_ and CuAl_10_Ni_5_Fe_4_-0.20% Be alloys.

**Figure 8:**
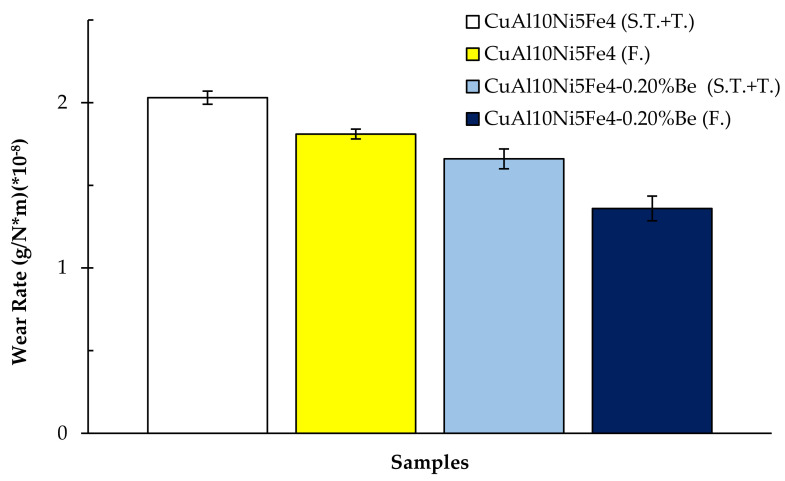
Wear rates of CuAl_10_Ni_5_Fe_4_ and CuAl_10_Ni_5_Fe_4_-0.20% Be alloys after wear.

**Figure 9:**
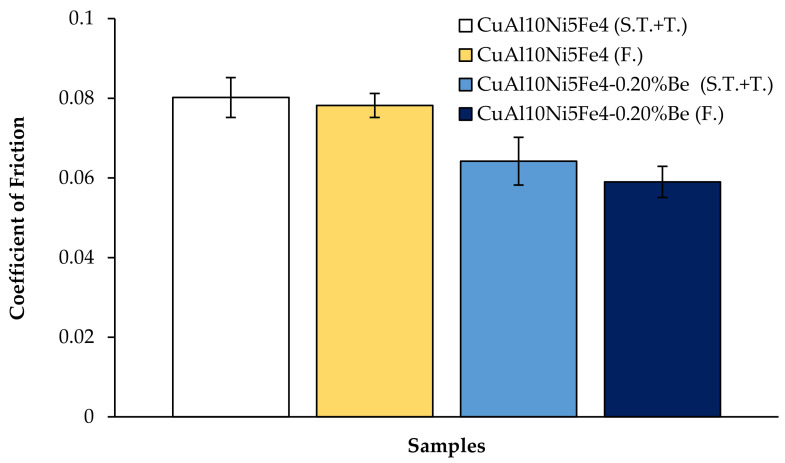
Post-wear friction coefficients of CuAl_10_Ni_5_Fe_4_ and CuAl_10_Ni_5_Fe_4_-0.20% Be alloys.

Figure 7 shows the weight losses after the wear of the CuAl_10_Ni_5_Fe_4_ and CuAl_10_Ni_5_Fe_4_-0.20% Be alloys after solution treatment (ST) + tempering (T) and forging (F) processes, sliding at a speed of 0.1 m/s and covering a total distance of 10,000 m. The calculated wear rates of the alloys after this wear are given in Figure 8. The friction coefficients evaluated during the wear are shown in Figure 9. The alloy with CuAl_10_Ni_5_Fe_4_-0.20% Be added showed the best wear behavior after solution treatment (ST) + tempering (T) and forging (F). After forging (F), the CuAl_10_Ni_5_Fe_4_-0.20% Be alloy had a weight loss of 0.002723334 g and a wear rate of 1.36167 × 10^−8^ g/N*m at the end of 10,000 m, while the friction coefficient was 0.059. The incorporation of beryllium into CuAl_10_Ni_5_Fe_4_ enhances the alloy’s resistance. The relationship between the hardness and wear performance is essential, as harder materials often have superior wear properties, therefore prolonging their lifespan under challenging conditions [52]. The wear characteristics of CuAl_10_Ni_5_Fe_4_ are markedly affected by the existence and properties of its κ-phases. The κ-phases, intermetallic compounds generated during the alloying process, are essential in influencing the mechanical characteristics and wear resistance of CuAl_10_Ni_5_Fe_4_ [45,53]. The selective corrosion behavior suggests that the κ-phases might offer a protective advantage against wear under specific conditions, thereby prolonging the service life of CuAl_10_Ni_5_Fe_4_ components. Furthermore, the mechanical characteristics of CuAl_10_Ni_5_Fe_4_, especially its wear resistance, are influenced by the heat treatment operations it undergoes. Heat treatments can modify the distribution and morphology of the κ-phases, therefore affecting the alloy’s hardness and wear resistance [4,7,54]. The microstructural alterations caused by forging and subsequent heat treatments are crucial in influencing the hardness of CuAl_10_Ni_5_Fe_4_. Heat treatment techniques can induce phase changes that improve the mechanical characteristics of the alloy. The lamellar structure in CuAl_10_Ni_5_Fe_4_ may be modified via equal-channel angular pressing followed by isothermal heat treatment, leading to a finer microstructure that enhances hardness [55,56]. The increased hardness resulting from forging can enhance wear resistance, which is crucial for components exposed to abrasive conditions [57]. Studies demonstrate that an increase in a material’s hardness often enhances its wear resistance. This relationship is obvious in studies that show reductions in the wear loss and wear rate with increasing hardness [58,59,60]. The AlFe_3_ phase, being a hard intermetallic compound, contributes to these phenomena by offering a resilient microstructure capable of enduring abrasive wear processes. Microstructural factors, such as the size and distribution of hard phases, like AlFe_3_, considerably affect the abrasive wear characteristics of the material [60,61]. Wear processes may differ based on the material’s phase composition. The inclusion of AlFe_3_ can modify the wear behavior from abrasive to adhesive wear, contingent upon the loading circumstances and the interaction with counter surfaces [62]. The AlNi intermetallic phase is recognized for its mechanical qualities, exhibiting relatively high hardness values that enhance wear resistance [63]. Moreover, the development of AlNi intermetallic phases has been associated with enhanced wear and friction properties [64]. The microstructural development of alloys with AlNi phases can substantially influence their tribological performance. It was shown that the coarsening of AlNi precipitates during heat treatment might result in diminished wear resistance, highlighting the need for microstructural management in preserving favorable mechanical characteristics [65]. The hardness of intermetallic phases, particularly those in high-entropy alloys, is essential for assessing the material’s overall wear behavior [66]. The hardness of materials with the AlCu_4_ phase is mainly because of the strengthening processes linked to the intermetallic compounds created during solidification and subsequent thermal treatments. The inclusion of hard phases, like AlCu_4_, can obstruct dislocation migration, thereby enhancing the alloy’s hardness. This is especially pertinent for wear resistance because tougher materials often demonstrate reduced wear rates in harsh environments. The hardness of these alloys can fluctuate considerably based on the phase composition and manufacturing techniques, which directly influence their wear performance [52,67]. Furthermore, the wear resistance of aluminum alloys comprising the AlCu_4_ phase can be improved in many ways. The intermetallic phases can serve as impediments to wear, diminishing material loss during tribological interactions. The development of a hard phase inside the matrix has been demonstrated to enhance wear resistance by offering a protective barrier against abrasive wear [67]. Beryllium addition influences the microstructural characteristics significantly. In copper–beryllium alloys, the microstructure typically consists of a face-centered-cubic (FCC) α-phase matrix with precipitates that can enhance mechanical properties through age-hardening mechanisms [16]. Beryllium promotes the formation of fine precipitates that can impede dislocation movement, thereby enhancing the strength of the alloy [21]. Moreover, studies have shown that beryllium can facilitate the formation of a more uniform microstructure, which is crucial for achieving desired mechanical and tribological properties [16]. The presence of beryllium leads to the formation of a solid solution that can refine the grain structure, thereby improving the overall strength of the alloy [68]. Specifically, the microstructure of CuAl_10_Ni_5_Fe_4_ with beryllium is expected to exhibit a distribution of intermetallic phases, which contribute to the alloy’s tribological performance [10]. The presence of intermetallic phases, such as Al_8_BeFe_2_Si, can enhance the mechanical properties of the base material, leading to improved wear performance. For instance, studies have shown that the addition of certain intermetallic phases can increase hardness and thermal stability, which directly correlate with enhanced friction and wear resistance [69]. Specifically, the Al_8_BeFe_2_Si phase can contribute to the formation of a more robust microstructure, which is crucial in resisting wear under various loading conditions. The presence of a second phase can lead to a more complex wear mechanism, where the wear rate is not solely dependent on the hardness of the material but also on the distribution and morphology of the phases present [70]. Moreover, the microstructural evolution induced by the presence of the Al_8_BeFe_2_Si phase can alter the wear mechanisms at play. The hardening effect of such phases can lead to a shift in the dominant wear mechanism from adhesive wear to abrasive wear, as the harder phases resist deformation and material removal more effectively [71]. This transition is critical in applications where wear resistance is paramount, as it can prolong the lifespan of components subjected to severe tribological conditions. The presence of intermetallic compounds, such as Be_4_Al, can significantly influence the tribological performance of materials [72]. Research has shown that the microstructure of alloys, including the presence of specific phases, like Be_4_Al, can lead to variations in wear behavior. For example, the mechanical and tribological properties of rapidly solidified NiAl alloys are adversely affected by the softening of phases at elevated temperatures, which results in increased wear rates [73]. This indicates that the stability and hardness of the phases present in the alloy are crucial for maintaining low wear rates under operational conditions. Moreover, the phase variation in materials can have pronounced effects on friction coefficients and wear rates. Studies have demonstrated that different phase compositions can lead to significant changes in the tribological performance of materials, with certain phases providing better lubrication and wear resistance than others [74].

The wear performances of aluminum bronze alloys, particularly those with beryllium (Be) additions, can be compared to those of other well-known copper alloys, such as tin bronzes and brasses. Aluminum bronze alloys are recognized for their excellent wear and corrosion resistances, which are attributed to their unique microstructures and the presence of aluminum, which enhance hardness and strength [51,75]. However, the addition of Be can further improve these properties, making aluminum bronze alloys particularly suitable for demanding applications in marine, aerospace, and automotive industries [76]. The wear performance of aluminum bronze alloys is significantly influenced by their microstructural characteristics. For instance, the presence of intermetallic compounds and the distribution of aluminum within the copper matrix can affect the wear resistance. Research indicates that aluminum bronzes with higher aluminum contents may exhibit increased brittleness, which can adversely affect their wear performances under high-load conditions [77]. Conversely, the incorporation of Be has been shown to enhance the mechanical properties of aluminum bronzes, leading to improved wear resistances compared to those of traditional copper alloys, like tin bronzes and brasses [78]. In comparison, tin bronzes, which typically contain tin as the primary alloying element, exhibit good wear resistances but may not match the performances of aluminum bronzes with Be additions. Tin bronzes are often used in applications requiring good machinability and corrosion resistance, but they can suffer from lower hardness and strength compared to those of aluminum bronzes [79]. Furthermore, brasses, which are primarily copper–zinc alloys, generally provide good ductilities and corrosion resistances but tend to have lower wear resistances compared to those of aluminum bronzes, particularly under abrasive conditions [80]. The tribological behaviors of these alloys are also influenced by their surface characteristics. Aluminum bronzes, especially those treated with advanced techniques, such as thermal spraying, can develop surface coatings that enhance their wear resistances [81]. In contrast, although brasses and tin bronzes can also be treated to improve their surface properties, they may not achieve the same levels of wear performance as aluminum bronzes with Be additions because of inherent differences in their microstructures and mechanical properties [51,75].

The coefficient of friction (CoF) is an essential factor in assessing the tribological performances of nickel–aluminum bronze (CuAl_10_Ni_5_Fe_4_) alloys, particularly in applications where wear resistance and durability are crucial, such as under maritime conditions. The coefficient of friction affects both the wear rates of materials and their overall performances in mechanical systems. Numerous studies have shown that the coefficient of friction (CoF) of CuAl_10_Ni_5_Fe_4_ may be influenced by multiple aspects, including surface treatments, load conditions, and environmental variables [82]. Furthermore, enhancing the wear resistance and coefficient of friction of nickel-containing aluminum bronzes underscores the significance of the alloy composition in influencing tribological parameters [15]. Research indicates that the friction coefficient of aluminum bronze is influenced by the microstructural characteristics imparted by the addition of beryllium [82,83,84]. This suggests that microstructural modifications due to beryllium addition can lead to improved wear resistance and lower friction coefficients. Additionally, the incorporation of beryllium can modify the mechanical interlocking at the interface, as noted by Kuang et al. in their examination of interfacial strength in composites [85]. The roughness introduced by beryllium particles can increase the bonding area, thereby enhancing the mechanical properties and potentially lowering the friction coefficient during sliding contact. This is corroborated by the findings of Morales, who discusses the wear mechanisms of aluminum bronzes and highlights the importance of the alloy composition in determining the tribological performance [86].

**Figure 10:**
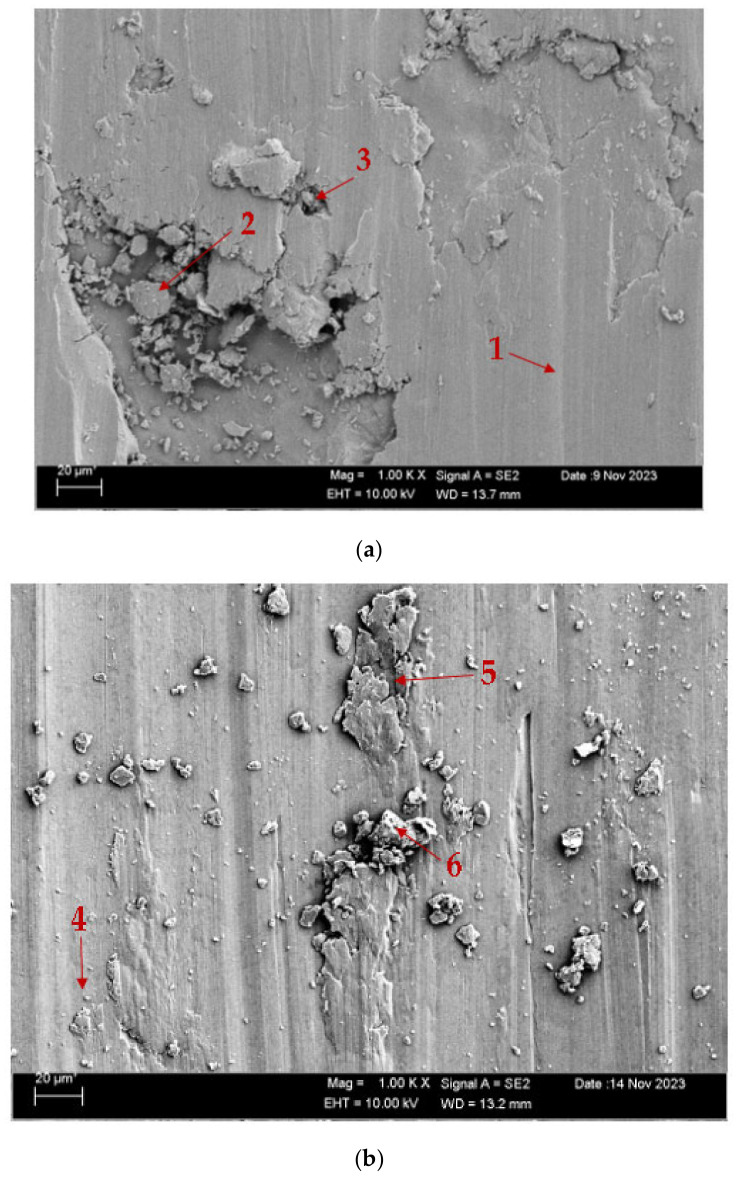
SEM images of wear after ST and T after casting: (**a**) CuAl_10_Ni_5_Fe_4_ and (**b**) CuAl_10_Ni_5_Fe_4_-0.20% Be.

**Figure 11:**
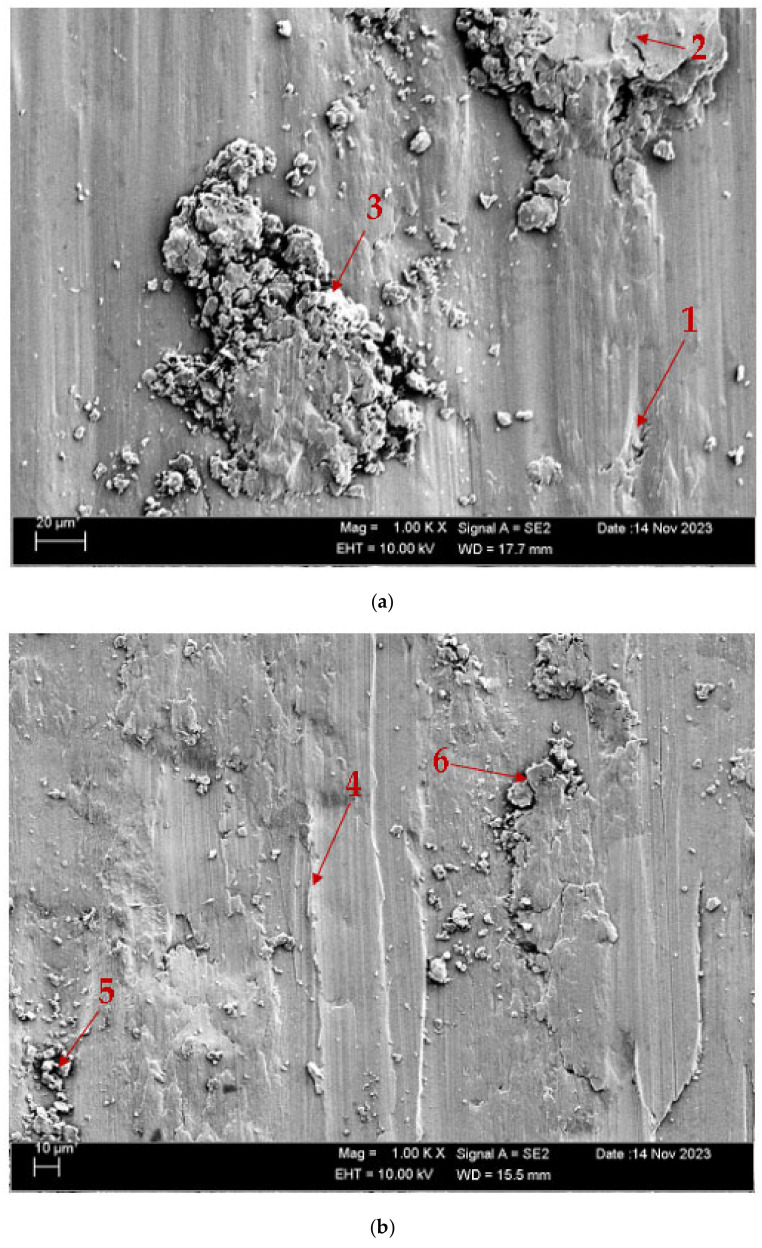
SEM images of wear after F: (**a**) CuAl_10_Ni_5_Fe_4_ and (**b**) CuAl_10_Ni_5_Fe_4_-0.20% Be.

Figure 10 shows the SEM images of the CuAl_10_Ni_5_Fe_4_ and CuAl_10_Ni_5_Fe_4_-0.20% Be alloys after solution treatment (ST) and tempering (T) after casting, while Figure 11 shows the SEM images of these alloys after forging (F). Table 4 and Table 5 show the EDX analyses of the worn debris exhibiting various morphologies, taken from the arrowhead points labeled as (1–6), as illustrated in the SEM images in Figure 10a,b and Figure 11a,b, respectively. According to SEM micrographs, the effective wear mechanisms are abrasive and adhesive wear types. According to the obtained wear micrographs, accumulations and wear grooves were formed around the wear track because of plastic deformation, and thin and medium linear wear tracks were formed as a result of abrasive wear. In addition, two-body and three-body wears were observed. In addition, adhesive wear, which involves material transfer between surfaces because of adhesion forces during sliding or contact, was frequently observed in the CuAl_10_Ni_5_Fe_4_ and CuAl_10_Ni_5_Fe_4_-0.20% Be alloys after forging. In the CuAl_10_Ni_5_Fe_4_ alloy, the dimensions of the parts broken off as a result of wear after both solution treatment (ST) and tempering (T) after casting and after forging are remarkable. The sharpness of the wear marks gradually decreased with the addition of the beryllium. In the CuAl_10_Ni_5_Fe_4_ alloy worn after solution treatment (ST) and tempering (T), very small oval wear pieces adhering to the wear marks are seen at point 1 in Figure 11a. At point 2, it is seen that the part broken off from the surface of the material, because of the wear effect, has formed a groove on the surface and large-sized residues. At point 3, wear marks are seen as residues accumulated in smaller cavities compared to those at point 2. In the CuAl_10_Ni_5_Fe_4_-0.20% Be alloy worn after solution treatment (ST) and annealing (T) after casting, it is seen, at point 4 in Figure 10b, that wear debris adheres to the wear marks due to the mechanical effect. At point 5, there was a crater-shaped piece plastered on the surface. At point 6, in addition to a situation similar to that at point 5, small wear pieces adhered to the surface. In Figure 11a, it is seen that a small circular piece that broke off in the wear track of the CuAl_10_Ni_5_Fe_4_ alloy worn after forging adhered to the surface. At point 2, large and small pieces that broke off as a result of wear adhered to the surface. At point 3, small circular wear pieces that adhered around a plastering similar to that at point 2 were seen. In Figure 11b, broken pieces plastered to the very thin wear tracks at point 4 of the CuAl_10_Ni_5_Fe_4_-0.20% Be alloy worn after forging are seen. At point 5, small grouped debris is seen plastered to the matrix surface. At point 6, small and dense debris and shells are plastered to the surface, like moss.

## 4. Conclusions

In this study, it was investigated how the tribological and microstructural properties of 0.20% beryllium (Be)-added CuAl_10_Ni_5_Fe_4_ alloys were affected by post-casting heat treatment (solution treatment (ST) and tempering (T)) and forging (F) methods. The results are as follows:

- The addition of 0.20% beryllium to the CuAl_10_Ni_5_Fe_4_ alloys resulted in an increase in the thickness and the needle-like shape of the dendritic arms formed by α-phases, as well as increases in the amounts of certain κ-phases. Furthermore, significant κ_IV_- and κ_II_-phase precipitation was detected in the tempered β-phase. The thickness of the microstructures in the CuAl_10_Ni_5_Fe_4_ and CuAl_10_Ni_5_Fe_4_-0.20% Be alloys significantly decreased during the forging process. The alloys maintained the same α-, β-, and κ-phases in the forging procedure. Because of the forging process, the dendritic arms underwent fracturing and were subsequently converted to almost spherical forms. In addition, the forging process resulted in the creation of additional κ_IV_-phases. The addition of 0.20% beryllium to the CuAl_10_Ni_5_Fe_4_ alloy resulted in the formation of distinct κI- and κ_III_-phases in the alloys;
- Although forging greatly increased the hardness values of both alloys, the hardness of the CuAl_10_Ni_5_Fe_4_ alloy without the beryllium addition was observed to be lower. Considering the difference in the hardness values after solution treatment (ST) and tempering (T) after casting and after forging (F), it was observed that the CuAl_10_Ni_5_Fe_4_-0.20% Be alloy showed the highest values of 235.29 HB and 255.08 HB, respectively;
- The alloy with CuAl_10_Ni_5_Fe_4_-0.20% Be added showed the best wear behavior after solution treatment (ST) + tempering (T) and forging (F). After forging (F), the CuAl_10_Ni_5_Fe_4_-0.20% Be alloy had a weight loss of 0.002723334 g and a wear rate of 1.36167 × 10^−8^ g/N*m at the end of 10,000 m, while the friction coefficient was 0.059.

## Figures and Tables

**Table 1:** Alloy weight percentages.

	Chemical Composition (wt.%)				
Alloy	Al	Fe	Ni	Be	Cu
CuAl_10_Ni_5_Fe_4_	10.19	3.01	4.41	-	Bal.
CuAl_10_Ni_5_Fe_4_-0.20% Be	9.99	2.99	4.38	0.2	Bal.

**Table 2:** EDX weight percentages at points in Figure 4a,b.

Point	Al	Be	Mn	Fe	Ni	Cu
1	6.65	-	0.25	0.41	5.38	87.32
2	6.12	-	0.20	0.17	4.97	88.55
3	7.47	-	0.32	2.20	5.93	84.09
4	5.53	1.05	0.16	0.38	5.19	87.71
5	10.76	0.46	0.06	0.41	8.37	79.95
6	6.57	0.81	0.61	2.41	7.03	82.59

**Table 3:** EDX weight percentages at points in Figure 5a,b.

Point	Al	Be	Mn	Fe	Ni	Cu
1	6.53	-	0.24	0.98	5.87	86.38
2	6.21	-	-	0.49	5.55	87.75
3	6.12	-	-	0.10	5.09	88.69
4	7.06	1.35	0.53	1.82	6.18	83.06
5	10.43	1.62	0.37	3.42	9.45	74.71
6	9.27	1.81	0.32	2.69	7.68	78.23

**Table 4:** EDX weight percentages at points in Figure 10a,b.

Point	C	O	Al	Be	Mn	Fe	Ni	Cu
1	2.64	3.32	7.23	-	0.89	0.21	6.13	79.60
2	3.06	5.60	6.62	-	0.80	2.21	6.04	75.65
3	3.36	6.14	6.27	-	0.78	1.65	5.72	76.10
4	2.09	2.61	8.14	0.80	0.23	3.44	7.67	75.04
5	2.29	6.57	7.08	0.54	0.33	2.31	6.43	74.44
6	3.80	5.42	9.10	1.02	0.11	2.29	5.83	72.45

**Table 5:** EDX weight percentages at points in Figure 11a,b.

Point	C	O	Al	Be	Mn	Fe	Ni	Cu
1	4.08	2.87	7.72	-	0.42	2.35	6.78	75.79
2	3.76	4.41	7.21	-	0.09	0.89	6.40	77.24
3	4.47	3.69	6.87	-	0.20	0.95	7.23	76.60
4	3.27	2.35	7.52	1.59	0.30	1.56	6.52	76.91
5	4.30	8.24	7.04	1.54	0.20	0.78	6.09	71.80
6	3.98	6.44	6.94	0.87	-	1.03	6.44	74.30

## Data Availability

The raw data supporting the conclusions of this article will be made available by the authors on request.

## References

[1] González Parra J.R., Covelo A., Pingarrón A., Hernandez M. (2023). Electrochemical Polarization as a Sustainable Method for the Formation of Bronze Patina Layers on a Quaternary Copper Alloy: Insight into Patina Morphology and Corrosion Behaviour. Sustainability.

[2] González L., Fontijn E. (2023). Unveiling the Legacy of Bronze Age Arrow Weapons in Southeast Asia: Function, Technology, and Cultural Significance. J. Ilmu. Pendidik. Dan. Hum..

[3] Özdoğan M. (2023). The Making of The Early Bronze Age in Anatolia. Old World J. Anc. Afr. Eurasia.

[4] Wu Z., Hu Q., Qin Z., Zhang Y., Xia D.-H., Hu W. (2021). Effect of Plastic Deformation on Mechanical Properties and Corrosion Resistance of Nickel-Aluminum Bronze. Anti-Corros. Methods Mater..

[5] Thossatheppitak B., Suranuntchai S., Uthaisangsuk V., Manonukul A., Mungsantisuk P. (2014). Microstructure Evolution of Nickel Aluminum Bronze Alloy during Compression at Elevated Temperatures. Adv. Mater. Res..

[6] Anantapong J., Suranuntchai S., Manonukul A., Uthaisangsuk V. (2014). Investigation of Nickel Aluminum Bronze Alloy under Hot Compression Test. Adv. Mater. Res..

[7] Böhm J., Linhardt P., Strobl S., Haubner R., Biezma M. (2016). Microstructure of a Heat Treated Nickel-Aluminum Bronze and Its Corrosion Behavior in Simulated Fresh and Sea Water. Mater. Perform. Charact..

[8] Chalasani D., Shalchi Amirkhiz B., Lloyd A., Ram G.D., Mohammadi M. (2020). Wire-Arc Additive Manufactured Nickel Aluminum Bronze with Enhanced Mechanical Properties Using Heat Treatments Cycles. Addit. Manuf..

[9] Ding D., Pan Z., Duin S., Li H., Shen C. (2016). Fabricating Superior NiAl Bronze Components through Wire Arc Additive Manufacturing. Materials.

[10] Öztürk S., Sünbül S., Metoğlu A., Önal S., İcin K. (2020). Characterisation of Nickel–Aluminium Bronze Powders Produced by the Planar Flow Casting Method. Mater. Sci. Technol..

[11] Tan Z., Guo Q., Zhai W., Zhao Z. (2012). Tribological Characteristics of Nickel-Aluminium Bronze CuAl10Ni5Fe4 against 30CrMnSiA Steel after the Prior Corrosion Treatment. Appl. Mech. Mater..

[12] Yaseen M., Mansoor M., Ansari H., Hussain S., Khan S. (2018). Effect of Heat Treatment on Tribological Characteristics of CuAl_10_Ni_5_Fe_4_ Nickel Aluminum Bronze. Key Eng. Mater..

[13] Atapek S., Aktaş G., Polat S., Pisarek B. (2017). Tribological Characterization of Al-Bronzes Used as Mold Materials. Arch. Foundry Eng..

[14] Podrabinnik P., Gershman I., Mironov A., Kuznetsova E. (2018). Mechanisms of Obtaining Secondary Structures on Friction Surface of Experimental Aluminum Alloys for Monometallic Journal Bearings. Lubricants.

[15] Kulakli A., Şeşen F., Çitrak T., Özeren T. Effect of Cobalt and Titanium Additions on Corrosion and Wear Resistance of Nickel Containing Aluminum Bronzes. Proceedings of the METAL Conference.

[16] Alisha S., Venkateswaran T., Amruth M., Pammi C., Sivakumar D. (2015). Effect of Heat Treatment on the Mechanical Properties of Copper-Beryllium Alloy (C17200). Mater. Sci. Forum.

[17] Khodabakhshi A., Abouei V., Mortazavi A., Razavi S., Hooshyar H., Esmaily M. (2015). Effects of Cold Working and Heat Treatment on Microstructure and Wear Behaviour of Cu–Be Alloy C17200. Tribol. Mater. Surf. Interfaces.

[18] Higuera Cobos O., Niño I., Orozco L., Ferrer S., Mesa D. (2018). Wear Behaviour of Copper-Beryllium Alloy under Abrasive Conditions. Contemp. Eng. Sci..

[19] Özkan D., Türküz C. (2020). Chromium Nitride-Coated Copper Beryllium as a Cam Tappet Material Candidate. Proc. Inst. Mech. Eng. Part C J. Mech. Eng. Sci..

[20] Alkmin L., Araújo Pinto da Silva A.A., Nunes C., Santos C., Coelho G. (2014). Microstructural Evidence of Beryllium in Commercial Dental Ni-Cr Alloys. Mater. Res..

[21] Wang Z., Li J., Zhang Y., Lv C., Li T., Zhang J., Hui S., Peng L., Huang G., Xie H. (2022). Comparison of the Mechanical Properties and Microstructures of QB2.0 and C17200 Alloys. Materials.

[22] Smith N., Kvithyld A., Tranell G. (2018). The Mechanism Behind the Oxidation Protection of High Mg Al Alloys with Beryllium. Metall. Mater. Trans. B.

[23] Smith N., Gleeson B., Saidi W., Kvithyld A., Tranell G. (2019). Effects of CO_2_ Cover Gas and Yttrium Additions on the Oxidation of AlMg Alloys.

[24] Osinskaya Y., Petrov S., Pokoev A., Radzhabov A., Runov V. (2012). Kinetics of Aging of the Cu-Be Alloy with Different Beryllium Concentrations in an External Constant Magnetic Field. Phys. Solid State.

[25] Huang Y., Li W., Wu M., Xiao D., Huang L., Liu W. (2023). Effects of Beryllium Addition on Microstructure and Performance of Al-Mg-Li Alloys. Materials.

[26] Peng C.-H., Hou P.-Y., Lin W.-S., Shen P.-K., Huang H.-H., Yeh J.-W., Yen H.-W., Huang C.-Y., Tsai C.-W. (2023). Investigation of Microstructure and Wear Properties of Precipitates-Strengthened Cu-Ni-Si-Fe Alloy. Materials.

[27] Tan Z., Guo Q., Li X., Zhao Z. (2011). The Tribological Behaviour of Beryllium Copper Alloy QBe2 against 30CrMnSiA Steel under Sliding Condition. Adv. Mater. Res..

[28] Wannaprawat N., Karuna T. (2020). Study of Deep Cryogenic Treatment Process Effect on Microstructure and Properties of CuBeZr Alloy. Key Eng. Mater..

[29] Anantapong J., Uthaisangsuk V., Suranuntchai S., Manonukul A. (2014). Effect of Hot Working on Microstructure Evolution of As-Cast Nickel Aluminum Bronze Alloy. Mater. Des..

[30] Lv Y., Wang L., Han Y., Xu X., Lu W. (2015). Investigation of Microstructure and Mechanical Properties of Hot Worked NiAl Bronze Alloy with Different Deformation Degree. Mater. Sci. Eng. A.

[31] Faria M., Moraes Leite M., Ichikawa R., Vichi F., Turrillas X., Martinez L. (2020). Thickness Estimation of TiO_2_-Based Nanotubes Using X-Ray Diffraction Techniques. Mater. Sci. Forum.

[32] Schuetzke J., Schweidler S., Münke F., Orth A., Khandelwal A., Breitung B., Aghassi-Hagmann J., Reischl M. (2023). Accelerating Materials Discovery: Automated Identification of Prospects from X-Ray Diffraction Data in Fast Screening Experiments. Adv. Intell. Syst..

[33] Daroonparvar M., Atabaki M., Vakilipour A. (2015). Effect of Pre-Heat Treatment on Corrosion Behaviour of Nickel-Aluminum Bronze Alloy. Metalurgija.

[34] Ding Y., Lv Y., Chen K., Zhao B., Han Y., Wang L., Lu W. (2018). Effects of Microstructure on the Stress Corrosion Cracking Behavior of Nickel-Aluminum Bronze Alloy in 3.5% NaCl Solution. Mater. Sci. Eng. A.

[35] Hårsta A., Rundqvist S. (1987). The Crystal Chemistry of Kappa-Phases. J. Solid State Chem..

[36] Pisarek B. (2013). Model of Cu-Al-Fe-Ni Bronze Crystallization. Arch. Foundry Eng..

[37] Ajeel S., Yaseen R., Kadhim A. (2019). Microstructure and Mechanical Properties of Homogenized Stir Casting Aluminum Bronze Alloys. Al-Qadisiyah J. Eng. Sci..

[38] Yin T., Zhang S., Zhou F., Huo R., Zhang C., Chen J. (2022). Effects of Heat Treatment on Microstructure and Wear Behavior of Modified Aluminum Bronze Coatings Fabricated by Laser Cladding. J. Mater. Eng. Perform..

[39] Zykova A., Panfilov A., Chumaevskii A., Vorontsov A., Nikonov S., Moskvichev E., Gurianov D., Savchenko N., Tarasov S., Kolubaev E. (2022). Formation of Microstructure and Mechanical Characteristics in Electron Beam Additive Manufacturing of Aluminum Bronze with an In-Situ Adjustment of the Heat Input. Russ. Phys. J..

[40] Wharton J., Stokes K. (2008). The Influence of Nickel–Aluminium Bronze Microstructure and Crevice Solution on the Initiation of Crevice Corrosion. Electrochim. Acta.

[41] Nabach W. (2003). The Effects of Isothermal Deformation and Annealing on the Microstructure of Nickel-Aluminum-Bronze Propeller Material. Bachelor’s Thesis.

[42] Barik R., Wharton J., Wood R., Stokes K. Nickel-Aluminium Bronze Pitting Corrosion in Seawater Environment and Mitigation. Proceedings of the EUROCORR Conference.

[43] Ding Y., Zhao R., Qin Z., Wu Z., Wang L., Lei L., Lu W. (2019). Evolution of the Corrosion Product Film on Nickel-Aluminum Bronze and Its Corrosion Behavior in 3.5 Wt% NaCl Solution. Materials.

[44] Iannuzzi M., Vasanth K., Frankel G. (2017). Unusual Correlation between SKPFM and Corrosion of Nickel Aluminum Bronzes. J. Electrochem. Soc..

[45] Zheng J., Liu R., Ning L. (2022). Selective Corrosion of Cast Nickel–Aluminum Bronze in Seawater. Mater. Corros..

[46] Maximov J., Duncheva G., Anchev A., Dunchev V., Argirov Y., Todorov V., Mechkarova T. (2022). Effects of Heat Treatment and Severe Surface Plastic Deformation on Mechanical Characteristics, Fatigue, and Wear of Cu-10Al-5Fe Bronze. Materials.

[47] Segun B., Chukwulozie O., Adeleye S. (2024). Effect of Tin Addition on the Mechanical Properties and Microstructure of Aluminium Bronze Alloyed with 4% Nickel. Eur. J. Theor. Appl. Sci..

[48] Oluwadare B.S., Adebayo A., Stephen J.T. (2019). The Influence of the Addition of Nickel on the Structure and Mechanical Properties of Aluminium Bronze Alloy. Rev. Ind. Eng. Lett..

[49] Mouda P.A., Azeez A., Hydershah S.J. (2016). Sliding Wear Behavior of Cryogenic Treated Copper Beryllium Alloy. Appl. Mech. Mater..

[50] Kaczynski D. (2002). Beryllium, Beryllium Alloys, and Beryllium Composities.

[51] Nascimento M., Santos G., Teram R., Torres dos Santos V., Silva M., Couto A. (2019). Effects of Thermal Variables of Solidification on the Microstructure, Hardness, and Microhardness of Cu-Al-Ni-Fe Alloys. Materials.

[52] Çetin T., Akkaş M. (2020). Effect of WC Reinforced on Microstructure and Mechanical Properties of Cualmn Alloys Produced by Hot Pressing Method. Eur. J. Tech..

[53] Zhang B.B., Wang J.Z., Yuan J.Y., Yan F.Y. (2017). Tribocorrosion Behavior of Nickel Aluminum Bronze in Seawater: Identification of Corrosion-wear Components and Effect of pH. Mater. Corros..

[54] Anene F., Nwankwo N., Nwoke V. (2019). Effect of Dopant and Heat Treatment on the Microstructure and Mechanical Properties of Nickel-Aluminum Bronze. Metall. Mater. Eng..

[55] Barr C., McDonald D., Xia K. (2013). Significantly Enhanced Tensile Strength and Ductility in Nickel Aluminium Bronze by Equal Channel Angular Pressing and Subsequent Heat Treatment. J. Mater. Sci..

[56] Dutta V., Thakur L., Singh B., Vasudev H. (2022). A Study of Erosion–Corrosion Behaviour of Friction Stir-Processed Chromium-Reinforced NiAl Bronze Composite. Materials.

[57] Berlanga-Labari C., Claver A., Biezma M., Fernandez Palacio J. (2023). Study of Effect of Nickel Content on Tribocorrosion Behaviour of Nickel–Aluminium–Bronzes (NABs). Lubricants.

[58] Rodinger T., Lukšić H., Ćorić D., Rede V. (2024). Abrasion Wear Resistance of Precipitation-Hardened Al-Zn-Mg Alloy. Materials.

[59] Zhang X., Xu J., He W., Jia J. (2024). Effect of Multidirectional Forging and Aging Treatment on Wear Properties of ZK61 Alloy. Materials.

[60] Jha A.K., Gachake A., Prasad B.K., Dasgupta R., Singh M., Yegneswaran A.H. (2002). High Stress Abrasive Wear Behavior of Some Hardfaced Surfaces Produced by Thermal Spraying. J. Mater. Eng. Perform..

[61] Chitturi V., Pedapati S.R., Awang M. (2020). Investigation of Weld Zone and Fracture Surface of Friction Stir Lap Welded 5052 Aluminum Alloy and 304 Stainless Steel Joints. Coatings.

[62] Kumar Gatenda C., Liu Y., Zhu W. (2023). Effect of W Content on Wear Resistance of CoCrFeNiMnWx High Entropy Alloy. Proceedings of the Smart Manufacturing and Material Processing (SMMP).

[63] Tang S., Li Y., Gao Y., Zheng Q., Liu Z., Ren X. (2018). First-Principles Investigations of the Structural, Anisotropic Mechanical, Thermodynamic and Electronic Properties of the AlNi_2_Ti Compound. Crystals.

[64] Jainulabdeen A., Ramanathan S., Vinoth S., Ganesan V., Priyadharshini S., Krishnakumar K. (2021). Studies on Microstructural Evolution and Wear Behaviour of AlNi Intermetallic Reinforced AA6061 Alloy in T6 Condition. Arch. Metall. Mater..

[65] Yan D., Shi C., Wang J., Zhang Y., Sun J., Wang Y., Liu P. (2023). Microstructure and Properties of Laser Cladding AlxFeCoCrNiMn High Entropy Alloy of Q345 Steel. Mater. Res..

[66] Ayyagari A., Hasannaeimi V., Grewal H., Arora H., Mukherjee S. (2018). Corrosion, Erosion and Wear Behavior of Complex Concentrated Alloys: A Review. Metals.

[67] Wang Y., Yang C., Li J., Wang Y., Liu F., Liang X., Sun X. (2020). Effects of TiC Formation on the Microstructure and Hardness of Wear-Resistant Steel. Mater. Sci. Technol..

[68] Sankar V., Arravind R., Dhayalan M. (2018). Material Synthesis, Characterization and Machining Performance of Stir Cast Beryllium Copper Alloy Composites. Trans. Can. Soc. Mech. Eng..

[69] Chen Q., Zhao Z., Zhu Q., Wang G., Tao K. (2018). Cerium Addition Improved the Dry Sliding Wear Resistance of Surface Welding AZ91 Alloy. Materials.

[70] Pędzich Z., Grabowski G., Saferna I., Ziąbka M., Gubernat A., Szczerba J., Bucko M., Kot M. (2014). The Abrasive Wear of Non-Oxide Structural Ceramics in Wet Environment. J. Mater. Sci. Chem. Eng..

[71] Wang K., du D., Liu G., Chang B., Hong Y. (2019). Microstructure and Properties of WC Reinforced Ni-Based Composite Coatings with Y_2_O_3_ Addition on Titanium Alloy by Laser Cladding. Sci. Technol. Weld. Join..

[72] Kim S.-H., Kim H., Kim N. (2015). Brittle Intermetallic Compound Makes Ultrastrong Low-Density Steel with Large Ductility. Nature.

[73] Sheng L.Y. (2016). Microstructure, Mechanical and Tribological Properties of the Rapidly Solidified NiAl/Cr(Mo,Dy) Hypoeutectic Alloy. Mater. Sci. Forum.

[74] Ranjan N., Kamaraj M., Sundara R. (2023). Nanoparticles Encapsulated Carbon Nanotubes Dispersed Lubricants for Enhanced Tribology through Particle-phase Variation. Lubr. Sci..

[75] Li W., Liu Y. (2011). Effect of Ce on Wear Behavior of Plasma Spray Welded Novel Aluminum Bronze Coatings. Adv. Mater. Res..

[76] Dias A., Rodrigues G., Mendonça C., Silva G. (2019). Analysis of the Densification of a Composite Obtained by Sintering Process of Aluminum Bronze Powders with Different Carbides. REM Int. Eng. J..

[77] Setyana L., Santoso N., Suharnadi B., Prayoga B.T., Wiyadi W. (2022). Bonding of Interface Bimetal Aluminum-Bronze for Bimetal Bushing Produced by Solid Liquid Method. Acta Metall. Slovaca.

[78] Hu Y., Zhao H., Zhang Y., Zhang B., Hu K. (2023). Enhanced Mechanical Properties of QAl9-4 Aluminum Bronze for High-Speed-Rail Brake Systems with a Pulsed Magnetic Field. Materials.

[79] Chobaomsup V., Tachai L. (2011). Effect of Aluminum Addition to Cu-10wt%Sn Bearing on Its Pin-on-Disc Wear Behavior. Adv. Mater. Res..

[80] Yan J., Lindo A., Schwaiger R., Hodge A.M. (2019). Sliding Wear Behavior of Fully Nanotwinned Cu Alloys. Friction.

[81] Miguel J.M., Vizcaino S., Lorenzana C., Cinca N., Guilemany J.M. (2011). Tribological Behavior of Bronze Composite Coatings Obtained by Plasma Thermal Spraying. Tribol. Lett..

[82] Chen C., Yang Q., Chen Q., Wang Y., Xu D., Li H., Xiliang Z., Harvey C., Liu J. (2022). Tribological Properties of Copper-Embedded Self-Lubricating Bearing Materials. Ind. Lubr. Tribol..

[83] Zhong L., Wei G., Wang G., He X., Liao Y., Xie N. (2019). Effects of Femtosecond Laser Texture on the Tribological Properties of 20CrNiMo/Beryllium Bronze Tribo-Pairs of Rock Bit Sliding Bearings under Non-Newtonian Lubrication. Proc. Inst. Mech. Eng. Part J J. Eng. Tribol..

[84] He X., Zhong L., Wang G., Liao Y., Liu Q. (2015). Tribological Behavior of Femtosecond Laser Textured Surfaces of 20CrNiMo/Beryllium Bronze Tribo-Pairs. Ind. Lubr. Tribol..

[85] Kuang Z., Xia Y., Chen G., Sun D., Ju B., Look J., Yang W., Wu G. (2023). Effect of Interfacial Strength on Mechanical Behavior of Be/2024Al Composites by Pressure Infiltration. Materials.

[86] Morales J., Piamba O., Olaya J., Vallejo F. (2024). Effect of Heat Treatment on the Electrochemical and Tribological Properties of Aluminum-Bronze Coatings Deposited Used the Thermal Spraying Process. Coatings.

